# Tolerability of gefitinib in patients receiving treatment in everyday clinical practice

**DOI:** 10.1038/sj.bjc.6601477

**Published:** 2003-12-10

**Authors:** N van Zandwijk

**Affiliations:** 1 Department of Thoracic Oncology and Biometrics, The Netherlands Cancer Institute, Amsterdam, The Netherlands

**Keywords:** gefitinib (‘Iressa’, ZD1839), EGFR-TKI, NSCLC, side effects, tolerability, risk:benefit

## Abstract

Gefitinib (‘Iressa’, ZD1839), an epidermal growth factor receptor tyrosine kinase inhibitor, has recently been approved in several countries for use in advanced or metastatic non-small-cell lung cancer (NSCLC). In contrast to chemotherapies, which are generally used at or near their maximum-tolerated dose (MTD), gefitinib is used at an optimal biological dose (250 mg day^−1^), which is substantially below its MTD, minimising the risk of adverse events without compromising efficacy. Tolerability data from the compassionate use of gefitinib in the ‘Iressa’ Expanded Access Programme support the favourable safety profile of the agent reported in Phase I and II trials. In both settings, the majority of adverse drug reactions were mild/moderate and consisted mainly of grade 1/2 diarrhoea and skin rash. Although skin rash has been suggested to predict response to gefitinib, available data do not support this hypothesis. Overall, these tolerability data demonstrate that gefitinib has a relatively benign side-effect profile and is a well-tolerated treatment option for patients with previously treated NCSLC, who currently have few alternatives.

In advanced non-small-cell lung cancer (NSCLC) platinum doublets are regarded standard for palliative therapy for patients of performance status (PS) 0–1, but their palliative use is hindered by the toxicity elicited by these combinations. Cisplatin induces nausea and vomiting in a majority of patients and is also associated with nephrotoxicity, neurotoxicity, ototoxicity and anaemia (BC Cancer Agency, 2003; British National Formulary, 2003). In a significant number of cases these toxic effects result in dose reduction or withdrawal of cisplatin and transfer of the patient to an alternative treatment. Less fit patients (PS ⩾2) with NSCLC are advised to avoid platinum-based treatments (Schiller *et al*, 2002) and frequently receive single-agent chemotherapies first line. Gemcitabine, vinorelbine and the taxanes are being used as single agents for this indication and, although much better tolerated than in combination with platinum agents, their use may also be complicated by side effects.

The approved second-line chemotherapy option for patients of PS 0–1 who either cannot tolerate or who fail first-line platinum-based treatment is docetaxel. The administration of docetaxel is associated with prominent side effects such as neutropenia, alopecia, leucocytopenia and nail changes (BC Cancer Agency, 2003; British National Formulary, 2003). Patients receiving chemotherapies need to be closely monitored to avoid complications and discontinuation of treatment.

The toxicity issues that surround the use of standard chemotherapy highlight the clinical need for novel anticancer agents that have a mode of action that is different from the indiscriminate action of cytotoxic chemotherapies. It is anticipated that such agents will combine targeted antitumour activity with improved tolerability over cytotoxic agents. Gefitinib (‘Iressa’, ZD1839), an epidermal growth factor receptor tyrosine kinase inhibitor (EGFR-TKI), is an example of a targeted agent that has demonstrated favourable tolerability and durable antitumour activity in clinical trials in patients with advanced NSCLC (Fukuoka *et al*, 2003; Kris *et al*, 2003). The assessment of the gefitinib safety data reported via the ‘Iressa’ Clinical Experience (ICE) meeting, held in June 2003 in Madrid, Spain, provided valuable insight into the tolerability of the agent in everyday clinical practice. This article summarises the safety and tolerability findings from this experience and assesses how they compare with the safety and tolerability conclusions from the pivotal Phase II studies (Fukuoka *et al*, 2003; Kris *et al*, 2003). We also explore the biological basis of the good tolerability of gefitinib and discuss the impact of the agent's side-effect profile on its risk:benefit ratio.

## TOLERABILITY OBSERVED IN IDEAL TRIALS

The tolerability of gefitinib 250 or 500 mg day^−1^ was assessed in two randomised, double-blind trials in 425 pretreated patients with advanced or metastatic NSCLC. The ‘Iressa’ Dose Evaluation in Advanced Lung cancer (IDEAL) 1 trial enrolled 209 patients from centres in Europe, Australia, South Africa and Japan, all of whom had received ⩽2 prior regimens, including one that was platinum based (Fukuoka *et al*, 2003). IDEAL 2 recruited 216 patients in the USA, who had previously received ⩾2 chemotherapy regimens, including a platinum compound and docetaxel, used either together or as separate regimens (Kris *et al*, 2003). The safety profile of gefitinib demonstrated in these trials was consistent with that reported in the Phase I trials, and no unexpected adverse drug reactions (ADRs) were observed. The most common ADRs were mild (grade 1 or 2) and consisted mainly of diarrhoea and skin rash, which typically presented during the first month of treatment. Although the profile of grade 1/2 ADRs was similar for both doses, grade 1/2 ADRs were more frequently reported with gefitinib 500 mg day^−1^. For example in IDEAL 2, grade 1/2 diarrhoea was reported in 56% of patients receiving gefitinib 250 mg day^−1^ and 69% of those receiving 500 mg day^−1^. Grade 3/4 ADRs were also dose related. In both trials, a higher incidence of grade 3/4 ADRs was reported in patients receiving gefitinib 500 mg day^−1^ than in those receiving the lower dose (23.6 *vs* 7.8%, respectively). Most of the ADRs were manageable and noncumulative. In both trials, few patients discontinued gefitinib due to ADRs (1.5% for 250 mg day^−1^ and 6.8% for 500 mg day^−1^). These data show that, although both doses of gefitinib were generally well tolerated, the 250 mg day^−1^ dose was better tolerated overall. As efficacy data from the IDEAL trials showed that both doses had similar antitumour activity, these data support 250 mg day^−1^ as the recommended dose for advanced NSCLC because it provides effective clinical benefit and retains favourable tolerability.

## TOLERABILITY OF GEFITINIB IN EVERYDAY CLINICAL PRACTICE

Centres participating in the ‘Iressa’ Expanded Access Programme (EAP) have administered gefitinib 250 mg day^−1^ to patients with advanced NSCLC on a compassionate basis. The Netherlands Cancer Institute case series is one of the largest groups of patients (*n*=100) from a single institution for which EAP experience is available (Haringhuizen, ICE abs; Haringhuizen *et al*, 2003). (See appendix for ICE abstracts). Results from this case series were discussed in detail at the ICE meeting. In this case series, >50% of patients presented with adenocarcinoma and stage IV disease (Table 1

**Table 1:** The Netherlands Cancer Institute EAP experience – patient demography (Haringhuizen *et al*, 2003)

Patients, *n*, evaluable/nonevaluable	92/8
Male/female, *n*	48/44
WHO/ECOG performance status^a^ 0/1/2/3/4	9/37/22/7/1
*Histology* (%)	
Adenocarcinoma	51
Squamous-cell carcinoma	20
Large-cell carcinoma	15
Bronchioalveolar carcinoma	6
Stage III/IV	13/79
*Previous chemotherapy, n*	
Chemonaive	7
First line	57
Second line	19
Third line	7
Fourth line	2

WHO, World Health Organization; ECOG, Eastern Cooperative Oncology Group.

a Not recorded in 16 patients.

). A substantial proportion of patients (approximately 33%) were unfit for chemotherapy and had PS ⩾2. The majority of patients (62%) had received one prior chemotherapy regimen and, of these, 94.1% had received platinum-based treatment. Consistent with the tolerability profile of gefitinib in clinical trials, the majority of adverse events experienced by these patients in this EAP study were mild (grade 1/2) and the most common grade 1/2 adverse effects were skin rash, diarrhoea and desquamation/itching (34.0, 21.8 and 21.8% of patients, respectively) (Haringhuizen, ICE abs). Grade 3/4 adverse events were rare and linked to skin and gastrointestinal adverse events (Tables 2

**Table 2:** The Netherlands Cancer Institute EAP experience – adverse events (*n*=92) (Haringhuizen, ICE abs)

	**Grade 1 (%)**	**Grade 2 (%)**	**Grade 3 (%)**	**Grade 4 (%)**
Diarrhoea	19.6	2.2	1.1	1.1
Skin rash	19.6	14.4	1.1	—
Desquamation/itching	20.7	1.1	1.1	—
Nausea/vomiting	14.1	4.4	—	—
Anorexia	13.0	10.9	—	—
Liver-enzyme elevation	3.3	2.2	—	—

and 3

**Table 3:** Safety and tolerability observations from eight case series >45 patients with advanced NSCLC who received gefitinib compassionately through the gefitinib EAP

**Author**	**Patients treated (evaluable), *n***	**Median age and/or range (years)**	**Histology/disease stage**	**Prior treatment experience**	**Overall assessment of gefitinib tolerability by author**
Haringhuizen, ICE abs	92 (92)	33–76	62% Adenocarcinoma	92% ⩾first-line chemotherapy	Good tolerability. Most side effects were mild. Most frequent side effects were grade 1/2 skin rash, diarrhoea and desquamation/itching (34.0, 21.8 and 21.8% of patients, respectively). ILD without symptoms observed in one (1%) patient, which resolved during treatment
			14% Stage III		
			86% Stage IV		
					
Gridelli [a], ICE abs	59 (57)		46% Adenocarcinoma	97% ⩾first-line treatment	Good tolerability in both elderly and unfit patients. Elderly group: the most frequent adverse events were grade 1/2 skin changes in five (28%) patients and grade 1 diarrhoea in three (17%) patients. PS ⩾2 group: the most frequent adverse events were grade 1 diarrhoea in two (5%) patients and grade 2 hypertransaminasaemia in one (3%) patient
	18 (18) (Elderly)	73.5	46% Squamous-cell		
	41 (39) (PS ⩾2)	60	2% Bronchioalveolar		
			7% Other		
			12% Stage IIIb		
			88% Stage IV		
					
Gridelli [b], ICE abs	83 (71)	33–80	47% Adenocarcinoma	98% ⩾first-line treatment	Good tolerability. The most frequent adverse events were grade 1/2 skin changes and grade 1 diarrhoea (11 and 10% of patients, respectively). Other reported adverse events: grade 2 hypertransaminasaemia in one (1%) patient and grade 1 epistaxis in one (1%) patient
			42% Squamous-cell		
			2% Bronchioalveolar		
			8% Other		
			13% Stage IIIb		
			87% Stage IV		
					
Bianco, ICE abs	49 (49)	59 (29–80)	49% Adenocarcinoma	100% ⩾second-line chemotherapy	Tolerability was excellent, two (4%) patients experienced mild, drug-related skin rash
	PS ⩽2		47% Squamous-cell		
			2% Bronchioalveolar		
			2% Large-cell		
			16% Stage IIIb		
			84% Stage IV		
					
Cortes-Funes, ICE abs	113 (113)	61 (36–83)	41% Adenocarcinoma	79% ⩾second-line chemotherapy	Well tolerated. Most side effects were mild/moderate. The most frequent adverse events were grade 1/2 skin toxicity 42.5% patients (3.5% grade 3/4), diarrhoea 21.2% patients (0.9% grade 3/4) and 20.4% asthenia (5.3% grade 3/4). Other adverse events included nausea and vomiting (grade 1/2 10.6%; grade 3/4 0.9%), anorexia (9.7%; 3.5%), neurological toxicity (9.7%; 1.8%) and pulmonary toxicity (0.9%; 0.9%)
			40% Squamous-cell		
			15% large-cell		
			4% Other		
			30% Stage I-IIa		
			70% Stage IIIb-IV		
					
Chioni, ICE abs	74 (72)	65 (43–81)	24% Adenocarcinoma	53% second-line chemotherapy	Well tolerated. Most side effects were mild: grade 1 diarrhoea (5.4%) and cutaneous toxicity (8%). Two patients had grade 3 diarrhoea (3%) and one patient had grade 3 diarrhoea plus cutaneous toxicity (1%)
			36% Squamous-cell		
			9% Bronchioalveolar		
			14% Undifferentiated large-cell		
			16% Other		
					
			Disease stage not reported		
de Braud , ICE abs	79 (67)	56 (31–77)	65% Adenocarcinoma	Median number of chemotherapy regimens=2 range (1–6)	Well tolerated. Most side effects were mild/moderate grade 1/2 skin reactions (30%), diarrhoea (9%) and nausea (4%). Grade 3/4 skin reactions occurred in 2% of patients
			18% Squamous-cell		
			4% Bronchioalveolar		
			5% Other		
			Locally advanced or metastatic disease		

a Case series were discussed at the ‘Iressa’ Clinical Experience (ICE) meeting and provided information on safety and tolerability. ILD=interstitial lung disease; EHP=Expanded Access Programme; NSCLC=non-small-cell lung cancer.

). Gefitinib was well tolerated by most patients and <6% of patients withdrew/discontinued treatment due to adverse events.

A further six case series, each consisting of >45 patients, were also submitted to the ICE meeting (Gridelli [a], ICE abs; Gridelli [b], ICE abs; Bianco, ICE abs; Cortes-Funes, ICE abs; Chioni, ICE abs; de Braud, ICE abs). Their safety findings are consistent with tolerability results from The Netherlands Cancer Institute case series (Haringhuizen, ICE abs) and are also summarised in Table 3.

**Figure 1:**
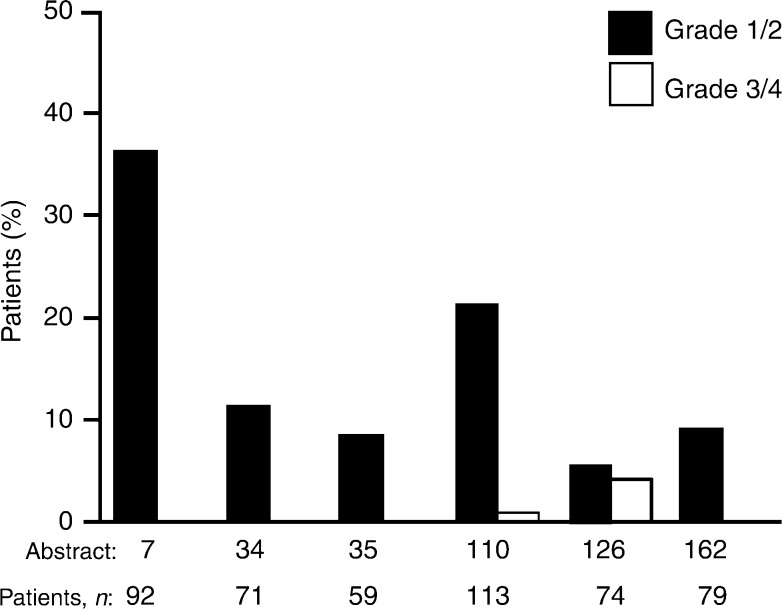
Incidence of diarrhoea in six case series with ⩾45 patients from the ICE meeting that reported diarrhoea as a commonly occurring adverse event (Haringhuizen, ICE abs; Gridelli [a], ICE abs; Gridelli [b], ICE abs; Cortes-Funes, ICE abs; Chioni, ICE abs; de Braud, ICE abs)

Overall, data from all these case series (Haringhuizen, ICE abs; Gridelli [a], ICE abs; Gridelli [b], ICE abs; Bianco, ICE abs; Cortes-Funes, ICE abs; Chioni, ICE abs; de Braud, ICE abs) (total *n* = 521) show that gefitinib is well tolerated by pretreated patients with NSCLC in everyday clinical practice. Most of the common adverse events were mild/moderate and the majority were grade 1/2 diarrhoea and skin rash (Figures 1

and 2

**Figure 2:**
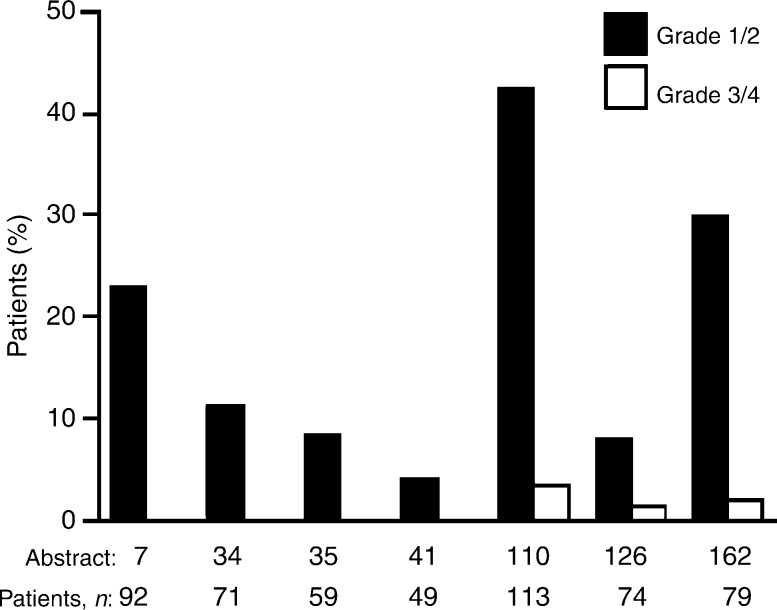
Incidence of skin rash in seven case series with ⩾45 patients from the ICE meeting that reported skin rash as a commonly occurring adverse event (Haringhuizen, ICE abs; Gridelli [a], ICE abs; Gridelli [b], ICE abs; Bianco, ICE abs; Cortes-Funes, ICE abs; Chioni, ICE abs; de Braud, ICE abs)

). Grade 3/4 adverse events were reported rarely (Table 3). Similar findings concerning the good tolerability of gefitinib in everyday clinical practice have also been reported by other large EAP case series in both adult (López Martin *et al*, 2003; Park *et al*, 2003) and elderly patients (Soto Parra *et al*, 2003).

Analysis of the safety data from gefitinib-treated patients has shown that unexpected or unusual adverse events with gefitinib are rare. One such rare event that has been recently featured in the media is interstitial lung disease (ILD), which was stimulated by the publication of a Japanese report of four patients who experienced interstitial pneumonia while receiving gefitinib (Inoue *et al*, 2003). ILD is not an uncommon event in patients who receive therapy for NSCLC and it may occur as a sign of progression of the disease itself (metastatic/lymphomytic spread) or as a consequence of its treatment. Standard lung cancer treatments, such as chemotherapy and radiotherapy, are able to elicit ILD, with incidences of 10% and higher reported (Chen *et al*, 2000; Thomas *et al*, 2000; Rebattu *et al*, 2001; Willner *et al*, 2001; Bhatia *et al*, 2002). ILD was not reported with gefitinib 250 mg day^−1^ in the IDEAL trials and has been reported rarely during its wider use in clinical practice. Four cases of ILD were reported at the ICE meeting (Haringhuizen, ICE abs; Overbeck and Griesinger, ICE abs; Cortes-Funes, ICE abs; Gervais, ICE abs). Analysis in over 92 000 patients worldwide, who have received gefitinib to date (September 2003), has shown that the incidence of ILD-type events was less than 1% (Forsythe and Faulkner, 2003). The frequency of ILD-type events in Japanese patients (1.9%) appears to be higher than in the rest of the world (0.3%); the reason for this is unknown, although it may be related to population or environmental differences or differences in clinical practice. Of interest, this observed ethnic difference in reporting rates does not extend to other South East Asian countries (eg China, Hong Kong, Korea, Malaysia, Philippines, Singapore, Taiwan and Thailand), where the frequency of reporting ILD is comparable to that in the rest of the world (0.3%). Further investigations to clarify the reasons for such an ethnic stratification in ILD incidence following treatment are underway.

Overall, these safety data from the EAP are consistent with the findings from the IDEAL trials, and confirm that gefitinib is a well-tolerated anticancer agent. Most of its side effects are mild and unusual or unexpected side effects are rare.

## BIOLOGICAL BASIS OF GOOD TOLERABILITY OF GEFITINIB

**Figure 3:**
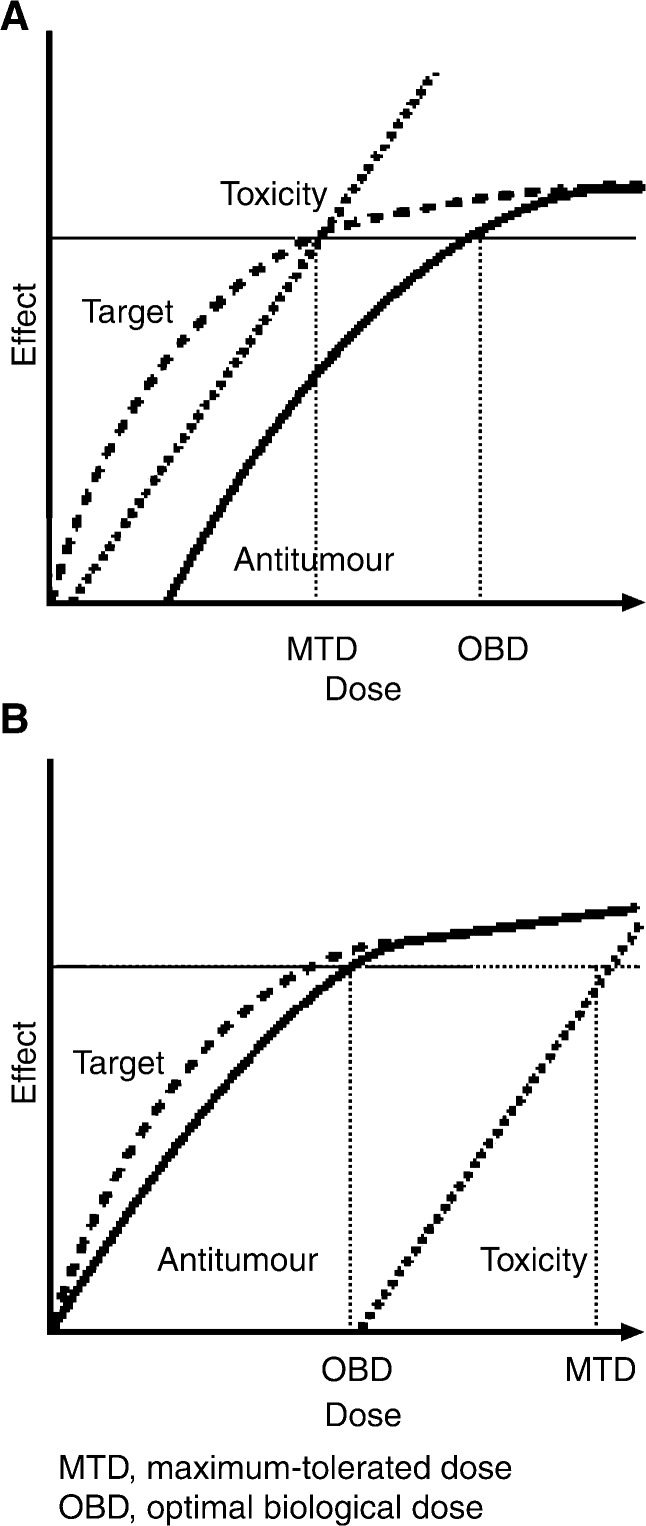
Idealised curves reflecting molecular target effects, antitumour effects and toxicity as functions for (**A**) a typical cytotoxic agent whose toxicity occurs at roughly the same dose as target effects and (**B**) a hypothetical target-based antiproliferative agent whose target effects occur at lower doses than toxic effects. Adapted with permission from: Rowinsky EK (2000)

Standard chemotherapeutic agents are cytotoxic drugs that kill dividing cells so their antitumour activity and toxic effects are generally seen within the same dose range. The dose of chemotherapy drugs selected for clinical use is usually the maximum-tolerated dose (MTD) (Figure 3A

). In contrast, as gefitinib is used at an optimal biological dose (OBD), which is substantially below its MTD, the risk of adverse events is minimised without compromising efficacy (Figure 3B) (Rowinsky, 2000). The pharmacodynamic differences between biologically targeted agents and standard chemotherapeutics may explain why gefitinib has a more favourable side-effect profile than some traditional chemotherapies.

**Figure 4:**
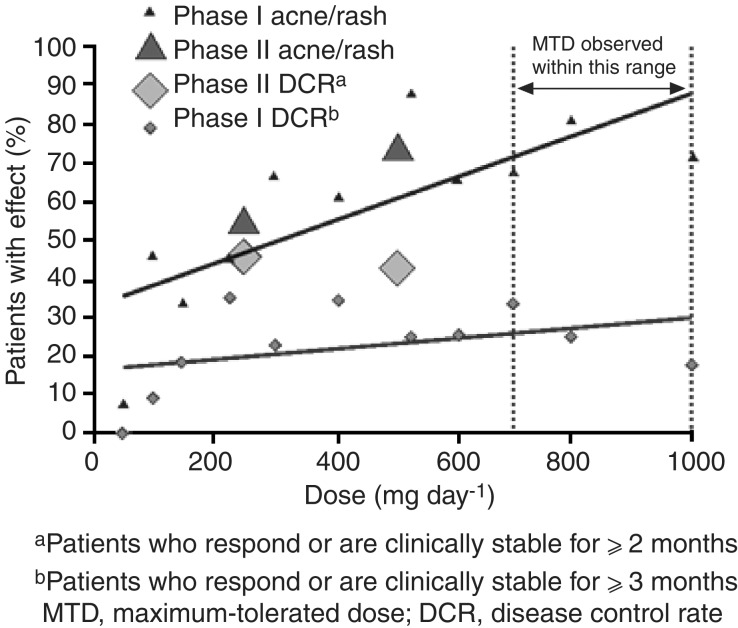
Relationship between gefitinib dose, objective response and rash. From: Herbst R (2003), with permission from Elsevier

Clinical evidence from Phase I and II trials has demonstrated that gefitinib has a wide therapeutic margin that enables it to be used at a dose that provides the optimal balance of efficacy and tolerability. Initial evidence relating to the OBD of gefitinib came from Phase I clinical trials, where the data suggested that gefitinib had biological and clinical activity over the complete dose range studied (150–1000 mg day^−1^) (Baselga *et al*, 2002; Herbst *et al*, 2002; Ranson *et al*, 2002; Nakagawa *et al*, 2003). On the basis of these data, two dose levels (250 and 500 mg day^−1^) were selected for Phase II/III studies, which were both significantly below the ⩾700 mg day^−1^ MTD of gefitinib identified in Phase I clinical trials (Ranson *et al*, 2002). Gefitinib 250 mg day^−1^ was chosen because it was above the lowest dose shown to effect antitumour activity and gefitinib 500 mg day^−1^ selected as it was the highest dose that was well tolerated by most patients during long-term use. Gefitinib was evaluated at these dose levels in the IDEAL trials and the data showed that both gefitinib 250 and 500 mg day^−1^ provided similar efficacy, but that the higher dose was associated with a higher frequency and severity of ADRs (Fukuoka *et al*, 2003; Kris *et al*, 2003) (Figure 4

).

### Skin rash and response

As detailed above, mild skin rash is one of the most common ADRs associated with gefitinib. Recent reports have suggested that skin rash may predict clinical response to EGFR inhibitors. These assumptions are based on the observations that most patients with a response, stable disease or long-term survival have a skin rash, there is a higher incidence of rash among responders than nonresponders and that survival end points are longer in patients with rash. These observations have been reported with the EGFR inhibitors gefitinib (Cohen *et al*, 2003; Janne *et al*, 2003), erlotinib (Perez-Soler *et al*, 2001) and cetuximab (Kies *et al*, 2002). However, as patients with an objective response generally receive treatment for longer than those with disease progression, it might be expected that there would be a higher incidence of rash in these patients, suggesting that simultaneous occurrence of rash and response may be coincidental. It is conceivable, however, that early-onset rash (one which develops within 14–28 days) may be more relevant to response than rash that develops later in treatment. To investigate this further, a retrospective analysis examined the onset of early rash in patients with NSCLC who received gefitinib in IDEAL 2 and survived ⩾28 days of treatment. Multivariate analysis found no statistically significant association between early-onset skin toxicity (skin rash, pruritus, acne, dry skin) and objective response rate with either gefitinib 250 or 500 mg day^−1^. By day 14, eight of the 12 patients (67%) who ultimately responded to gefitinib 250 mg day^−1^ had not developed any skin toxicity and by day 28, three of the 12 (25%) responders had not yet developed skin toxicity. Retrospective assessment of incidence of skin rash in both IDEAL trials has shown that nine of the 31 responders (29%) to gefitinib 250 mg day^−1^ did not experience skin rash at any time during treatment. Data from the gefitinib clinical trial programme have also shown that the incidence of skin rash, but not response, increases with increasing dose. In Phase I trials, in which gefitinib was tested over the dose range 150–1000 mg day^−1^, the incidence of rash correlated with escalating dose: from approximately 30% at 150 mg day^−1^ to approximately 80% at 1000 mg day^−1^ (Baselga *et al*, 2002; Herbst *et al*, 2002; Ranson *et al*, 2002; Nakagawa *et al*, 2003). Correlation between dose and skin rash was also evident in the Phase II IDEAL trials, where gefitinib 500 mg day^−1^ was associated with a higher incidence of rash but similar objective response rates and survival as the 250 mg day^−1^ dose.

These clinical data are supported by results from a study that analysed the pharmacodynamic effects of gefitinib in paired skin biopsies from patients who were receiving gefitinib 150–1000 mg day^−1^ in Phase I trials (Albanell *et al*, 2002). The study found that gefitinib inhibited skin EGFR activation at all doses ⩾150 mg day^−1^ and that there was no significant correlation between pharmacodynamic effects and skin toxicity.

Together, these clinical and experimental data do not support the hypothesis that skin toxicity predicts response to gefitinib.

## DISCUSSION

It is encouraging that the favourable safety profile of gefitinib demonstrated in Phase I and II trials is consistent with that observed in everyday settings. Data from clinical trials and the EAP indicate that gefitinib is well tolerated by patients with advanced or metastatic NSCLC. The majority of ADRs associated with gefitinib are mild in nature and those most commonly reported are grade 1/2 diarrhoea and skin reactions. Although skin rash has been hypothesised to be a potential prognostic factor for response to EGFR inhibitors, the lack of correlation between skin rash and clinical benefit over a wide range of gefitinib doses (150–1000 mg day^−1^) in clinical trials demonstrates that it should not be used to direct treatment with gefitinib. The analysis of trial data has shown that not all responders to gefitinib experience skin rash. Hence, it would seem inappropriate to use skin rash as a surrogate marker of response to gefitinib as it would deny patients who do not experience skin rash the potential to obtain benefit from treatment. Until more predictive molecular markers are identified, symptom improvement should be used as a meaningful prognostic indicator of the clinical benefit of gefitinib.

The relatively benign side-effect profile of gefitinib is very different from the safety profile of standard chemotherapy agents, with which patients frequently experience serious ADRs such as haematological toxicity, neurotoxicity, and nausea and vomiting. Often the severity of these ADRs requires medical intervention and in some cases patients will need to be hospitalised for management of toxicity caused by chemotherapy. In contrast, use of gefitinib in clinical trials and in everyday clinical practice shows that grade 3/4 ADRs and unexpected/unusual ADRs are rare. In addition, the findings show that the agent is not associated with cytotoxic ADRs and any nausea and vomiting experienced by patients during gefitinib treatment is generally mild to moderate.

It is suggested that pharmacodynamic differences between gefitinib and cytotoxic chemotherapies may account for gefitinib's favourable tolerability. Traditional chemotherapies are used at their MTD to exert their maximum efficacy but at the cost of a high level of toxicity and poor tolerability. In contrast, well-designed randomised dose-finding trials in NSCLC have demonstrated that gefitinib doses higher than 250 mg day^−1^ do not give a better response and cause increased toxicity. Hence, gefitinib is recommended to be used at an OBD (250 mg day^−1^) that provides clinical benefit while retaining favourable tolerability.

The contrasting safety profiles of gefitinib and chemotherapy highlight the important new approach that gefitinib brings to NSCLC. In addition to providing clinical benefit to a subset of patients with advanced or metastatic disease, the favourable safety profile of gefitinib avoids toxic reactions commonly seen with standard chemotherapy.
